# How the pandemic has changed innovation collaboration in SMEs, as illustrated by four co-innovation projects

**DOI:** 10.1177/14657503231190001

**Published:** 2023-07-24

**Authors:** Caroline Blais, Amélie Cloutier

**Affiliations:** Business School, 7321 Université de Sherbrooke, Sherbrooke, Canada; School of Management, 14845 Université du Québec à Montréal, Montreal, Canada

**Keywords:** product innovation, innovation collaboration, innovation process, small- and medium-sized enterprises (SMEs), pandemic, crisis

## Abstract

The recent health crisis has particularly affected small- and medium-sized enterprises (SMEs). Some have had to temporarily close their doors. Others chose to innovate by developing new products to take advantage of the situation and survive. However, innovation is a risky strategy, so there are many failures. Innovation collaboration is one way of limiting these failures and increasing the chances of project success. Nevertheless, little is known about SMEs’ innovation collaboration practices in the context of a crisis, and even before the pandemic, and further research is needed. This study aims to understand the innovation process and the collaboration practices adopted by SMEs for their product innovation projects to cope with the pandemic, by comparing their pre-pandemic practices with those implemented at the start of the pandemic. Based on four successful product innovation projects in two different SMEs, our results show that, at the start of the pandemic, collaborations included a larger number of partners, involved in more stages of the innovation process, in order to accelerate the new product development and commercialization, rapidly provide the necessary resources and response to customer needs.

## Introduction

Small- and medium-sized enterprises (SMEs) are particularly vulnerable to crises (Kraus et al., 2013) such as that generated by the COVID-19 pandemic. Crises are defined as ‘unplanned and unwanted [events] of limited duration and controllability with an ambivalent conclusion. They are capable of substantially and permanently endangering the continued existence of an entire company, or even making its existence no longer possible’ (Krystek, 1987: 6–7, cited by Kraus et al., 2013: 409). The health crisis, which forced the temporary closure of several SMEs, exacerbated the challenges they were already facing: financial fragility, labour shortages, and supply problems (Bartik et al., 2020). Furthermore, Eggers (2020) points out that quick and effective response to crises is a challenge for SMEs, due to their small size and limited resources.

In contrast, SMEs have certain characteristics – flexibility, rootedness in their local community, proximity to customers, agility – that give them unique capabilities to respond quickly and effectively to a crisis, seize opportunities and emerge stronger (Clauss et al., 2021; Dahles and Susilowati, 2015; De Waal and Knott, 2019). These characteristics also highlight their specific nature in innovation.

According to Wenzel et al. (2020), firms of any size can adopt one of four response strategies to a crisis: (1) retrenchment, which involves cutbacks of all kinds; (2) persevering, by maintaining the status quo; (3) innovating, by seizing emerging opportunities; and (4) exiting, or shutting down. A sudden and unexpected event, such as the pandemic, can represent an enormous development potential for SMEs, provided that their managers are open-minded and prepared to seek and seize innovation opportunities (Clauss et al., 2021). Our study focuses on SMEs that have adopted innovation as a response strategy to the COVID-19 crisis.

In the context of a crisis, SMEs may opt for innovation to ensure their survival and an adequate post-crisis recovery (Roper and Turner, 2020). Research and development (R&D) and innovation investments promote their survival and profitability (Roper and Turner, 2020). In fact, research shows a significant positive relationship between innovation and SMEs performance, viewed in terms of achieving targets related to sales, profits, asset capital, production and market share (Adam and Alarifi, 2021). Even in non-crisis situations, innovation represents an important factor in the survival and continuity of enterprises (Ortiz-Villajos, 2014). Innovation supports their expansion and enhances their success, making it an effective strategy for maintaining and improving their competitive advantages in more difficult economic circumstances (Chabbouh and Boujelbene, 2022). In the face of the pandemic, some SMEs sought to develop and commercialize new products to maintain their performance. They innovated in a hurry to ensure their survival and meet new market needs (Clauss et al., 2021).

Nevertheless, innovation is a double-edged sword: it increases the possibility of outstanding performance, but also of failure, for both SMEs and large firms (Buddelmeyer et al., 2010). Rhaiem and Amara (2019) estimate that between 40% and 90% of innovation projects fail, in whole or in part. This proportion is particularly high in the context of radical innovation, which makes the firm more financially vulnerable, especially when the managers lack experience or the employees have little expertise (Buddelmeyer et al., 2010).

Thus, SMEs must explore different avenues to innovate while minimizing risks. Innovation collaboration is one such avenue. To innovate successfully and develop new sources of income, SMEs sometimes collaborate with external partners and adopt a more open innovation (OI) strategy (Kraus et al., 2019). In the last decade, SMEs are relying less on internal new product development and more on collaboration with partners to overcome resource constraints and stimulate innovation (Classen et al., 2012). The ‘do-it-yourself’ mentality in innovation now seems outdated: firms are increasingly opting to pool resources and skills (Deniaud et al., 2016). As a result, innovation collaboration is a phenomenon that is attracting increased attention from both researchers and practitioners (Zahoor and Al-Tabbaa, 2020).

Guzzini et al. (2017) show that collaboration increases the innovation performance of firms. However, studies on innovation collaboration in the context of SMEs are scarce (Guertler and Sick, 2021). They are even more scarce when looking at innovation collaboration in a crisis context (apart from, e.g. Christos et al., 2016 and Markovic et al., 2021). Van Auken et al. (2021) invite researchers to further study innovation in a crisis context with more than one firm and compare it longitudinally with other contexts. Marzi et al. (2020) indicate that more research is needed to understand the deployment of SMEs’ innovation process and collaboration with partners and their impact on the success of their projects. This is especially true today, as the pandemic has imposed particular conditions for innovation. It is important to understand how crises influence the innovation process and the use of collaboration by SMEs.

The COVID-19 pandemic provides a unique context to study the response of SMEs in crises. Our study focuses on the innovation process and collaborative practices within four successful innovation projects, both pre- and post-pandemic, by answering the following research question: how has collaboration within the product innovation process in SMEs been modified by the pandemic? Based on two Canadian (province of Quebec) manufacturing SMEs that each successfully commercialized a new product innovation before the pandemic and another one at its beginning, our objective is to gain a better understanding of the innovation process and the collaborative practices adopted by these SMEs. Studying four completed product innovation projects allows for the comparison of the entire innovation process, from idea generation to commercialization, the type and number of collaborative innovation partners, and the intensity of this collaboration throughout the entire process.

The article is organized as follows. First, we describe the theoretical concepts and tools used to understand the phenomenon under study, followed by the methodology used to carry out our study. We then present and discuss the results in relation to the mobilized literature. Finally, we conclude by addressing the theoretical and managerial contributions, as well as the limits, of our study.

### Theoretical background

Our study bridges the gap between the literature on innovation, collaboration and the innovation process. The concepts mobilized are presented in five main parts: (1) product innovation; (2) product innovation process; (3) resource-based view (RBV) theory; (4) innovation collaboration and OI; and (5) collaboration partners. These concepts and theories are used to build an analysis grid that helps organize and interpret the results of our research. This grid, which facilitates the analysis process, is presented at the end of this section.

### Product innovation

The Oslo Manual is widely recognized and respected as a comprehensive international guideline for measuring and understanding innovation activities. It provides a standardized framework and terminology that enables meaningful comparisons and analysis across different countries and industries (Kunttu et al., 2021). By using the Oslo Manual, we aim to ensure consistency and alignment with existing literature and research in the field of innovation. Furthermore, the Oslo Manual's focus on collaboration and the different dimensions of the innovation process corresponds to the objective of our research. It also provides a solid basis for exploring the role of collaboration and its impact on successful product innovation in SMEs.

In its fourth edition, the Oslo Manual defines innovation as ‘a new or improved product or process (or combination thereof) that differs significantly from the unit's previous products or processes and that has been made available to potential users (product) or brought into use by the unit (process)’ (OECD, 2018: 20). In terms of novelty, innovation can be seen as ‘new to the firm only, new to the firm's market, or new to the world’ (OECD, 2018: 77) on a continuum from incremental to radical innovation. The conduct of projects, such as those related to product innovation, requires ‘a series of complex and interrelated activities’ (Um and Kim, 2018: 661).

### Product innovation process

Product innovation unfolds through a process in which a product is designed, developed, and commercialized to respond to a market opportunity, also known as the product innovation process (Krishnan and Ulrich, 2001).

The best-known model of the product innovation process is the Stage-Gate System (SGS) developed by Cooper (1990). This linear process model is broken down into five stages that are: (1) preliminary assessment; (2) detailed analysis; (3) product development; (4) testing and validation; and (5) commercialization. A pre-stage of ideation and five gates that serve as signals on whether to continue the process or not are added to these stages. A series of 13 activities, which are then broken down into several tasks, describe the work that needs to be done to develop a new product idea and bring it to market. The product innovation process, such as the one illustrated in the SGS model, is intended to facilitate the management of such projects (Sonta-Draczkowska and Mrozewski, 2019).

To meet the demands of today's market – shorter product lifetimes and tighter time-to-market – the innovation process must be flexible and fast in addition to adapting to customer needs that change more rapidly than in the past (Cooper, 2017; Magistretti et al., 2019). To accommodate these requirements, more succinct models, such as Stage-Gate Xpress or Lite (Cooper, 2008), and more agile and dynamic models, such as Agile Stage-Gate (Cooper and Sommer, 2016) are emerging, building on that the original SGS model. The agile, lite and Xpress versions derive from the need to accelerate product time-to-market but do not change the structure of activities and decisions made about continuing or abandoning a project (Cooper and Sommer, 2016).

According to Cocchi et al. (2021), SGS is often the primary innovation process model, sometimes hybridized with other methods to support more agile and rapid product development. A hybrid model can help manufacturing SMEs leverage the strengths of different methods – SGS and agile, for example – to reduce product development times and increase project success rates (Edwards et al., 2020). In a crisis context, this acceleration in product innovation becomes more important than ever (Cooper, 2021). Although sometimes criticized for being too static and linear, the SGS remains the most widely adopted product innovation process (Cocchi et al., 2021). Studies focusing on SMEs specify that they use several of the activities identified within SGS, but adapt the process to better meet their needs, especially in terms of flexibility (Huang et al., 2002; Leithold et al., 2015). Since it is the most comprehensive model in terms of stages, activities, tasks and gates, it provides a more complete picture of the innovation process adopted.

To innovate successfully and secure sufficient resources and skills, SMEs sometimes collaborate with partners on the development of new product ideas, even more in a crisis context (Blais, 2023). Collaboration contributes positively to the SMEs innovation process (Pierre and Fernandez, 2018), by accelerating product development, by reducing time-to-market (Morgan et al., 2019) and by accessing resources they may not have internally (El Hilali et al., 2020; Ledwith, 2000). SMEs are known to have limited resources and a narrow range of skills compared to large enterprises (van de Vrande et al., 2009). In this context, when studying innovation practices in SMEs, it is appropriate to use the precepts of RBV theory.

### RBV theory

The RBV theory (Penrose, 1959), according to which resources are the foundation of a firm's competitive advantage, is relevant for studying innovation collaboration in SMEs. This theory, widely accepted in strategic management (Newbert, 2007), is useful to our study for two main reasons. First, innovation collaboration by SMEs is generally motivated by the need to fill a resource gap (van de Vrande et al., 2009). Second, following Barney (1991), we conceive of the firm, our unit of analysis, as a ‘bundle of resources’. According to this theory, firms of all sizes, including SMEs, are composed of a set of resources. These can be tangible or intangible and include the firm's capabilities (in terms of R&D, knowledge, and learning). The need to access complementary resources can guide managers in choosing to engage in collaborative agreements.

The decision to collaborate depends on the resources available internally to the SME and the complementary resources available to a partner. This decision also lies in a needs and opportunities analysis (Arranz and Fdez. de Arroyabe, 2008). Thus, the organizational characteristics of the SMEs and their partner (resources and capabilities) are important factors in the formation of a collaborative relationship and the success resulting from it (Street and Cameron, 2007). Based on RBV, it can be conceived that collaborations provide SMEs with access to resources and skills required to innovate and obtain a sustainable competitive advantage (Lasagni, 2012).

### Innovation collaboration and open innovation

To classify the relative contributions of internal and external sources to innovation, the latter can range from imitation (item a) to developed mainly in-house (item e). In between, item b requires some internal innovation activities, item c requires considerable external input and item d external input as part of a collaboration with other organizations (OECD, 2018). These items can be altered, combined (items b and c) or disaggregated (item e) to identify the role of external sources and collaboration at a specific stage of the innovation process (OECD, 2018: 135):

- ‘Replicating products/business process already available from/to other firms or organizations, with no or very few additional changes by [a] firm.
- Developed by [a] firm by adapting or modifying products/business processes available from/to other firms or organizations, including reverse engineering.
- Developed by drawing substantially on ideas, concepts and knowledge sourced or acquired from other firms or organizations, directly or via intermediaries.
- Developed as part of a collaborative agreement with other firms or organizations, with all parties contributing ideas or expertise.
- Mainly developed by [a] firm on its own, from the idea to implementation’.

These distinctions between the contribution of internal and external sources pave the way for the concepts of cooperation, collaboration, and co-innovation, which have long been used undifferentiated in the literature (OECD, 2018). The term collaboration, chosen for our study, requires a great level of coordination (OECD, 2018: 134):

Collaboration requires coordinated activity across different parties to address a jointly defined problem, with all partners contributing. Collaboration requires the explicit definition of common objectives, and it may include agreement over the distribution of inputs, risks and potential benefits. Collaboration can create new knowledge, but it does not need to result in an innovation. Each partner in a collaboration agreement can use the resulting knowledge for different purposes.

Innovation collaboration is part of the OI trend which ‘denotes the flow of innovation-relevant knowledge across the boundaries of individual organizations. This includes proprietary-based business models that use [among others] collaborations […] to produce and share knowledge’ (OECD, 2018: 133). When collaboration between two or more partners results in innovation, this is known as co-innovation or coupled OI (OECD, 2018).

OI is a logical step for many SMEs to successfully innovate, develop new revenues and increase profitability (Kraus et al., 2019). OI changes the mindset around product innovation, shifting from R&D to a new way of developing a product from the combination of internal and external ideas and knowledge (Laviolette et al., 2016). External support is important for SMEs. It helps provide the knowledge needed to develop and implement innovations (Adam and Alarifi, 2021).

However, innovation collaboration involves challenges and even barriers. These include (a) loss of control over valuable knowledge; (b) high coordination costs; (c) loss of control over strategy; (d) difficulty finding the right partner; (e) difficulty establishing trust; (f) concerns about triggering antitrust policy enforcement, about employees leaking valuable information or know-how or about potential costs of dispute settlements; and (g) lack of time or financial resources (OECD, 2018). Trust therefore plays a crucial role in collaboration. Rousseau et al. (1998) define trust as ‘a psychological state comprising the intention to accept vulnerability based upon positive expectations of the intentions or behavior of another’. Trust also affects the level of formality of the collaboration, from formal to informal, with the formal level characterized by the presence of contracts. In SMEs, distinguishing this level of formality can be useful in understanding the deployment of innovation collaboration (Lu and Yu, 2020). In innovation, enterprises can collaborate with a wide variety of partners.

### Collaboration partners

The integration of collaboration partners can take place at various points during the innovation process. Such collaboration reduces technical and commercial risks and improves the chances of successful product innovation (Cooper, 2017; Deniaud et al., 2016).

Collaboration partners can be divided into five categories: (1) business enterprises (affiliated and unaffiliated), including suppliers, specialized knowledge service providers and commercial research institutes, customers and competitors/investors/other businesses; (2) government, including research institutes and other departments and agencies; (3) higher education; (4) private non-profit, including research institutes and other private non-profits; and (5) households/individuals (OECD, 2018: 138). These collaboration partners can be located at the local/regional level or elsewhere in the country (domestic) or abroad (OECD, 2018).

To explore the influence of the pandemic on collaboration in product innovation projects, we have developed an analytical framework known as the analysis grid presented in the next section. This framework serves as a bridge between the existing literature on innovation, collaboration in innovation (including collaboration partners) and the innovation process.

### Analysis grid

Guertler and Sick (2021) propose a grid for comparing open product innovation projects, including the following elements: objectives pursued, the team involved, previous collaboration experience, duration of the project, constraints, complications, obstacles, results and positive and negative aspects. The following grid (Table 1) combines concepts from Guertler and Sick (2021) with those of the Oslo Manual (OECD, 2018) and the innovation process (Cooper, 1990, 2008). It thus provides an overall picture of the elements to be observed in understanding innovation collaboration. The elements of comparison are categorized by project, partnership, process, and results. The analysis grid is used in the presentation of results in the SMEs’ collaboration practices before and at the beginning of the pandemic section.

**Table 1.** Grid for comparing collaboration in product innovation projects.

Categories and elements of comparison	References
**Project**	
Project name	Guertler and Sick (2021)
Objectives	Guertler and Sick (2021)
Team involved (SME)	Guertler and Sick (2021)
Duration of the project	Guertler and Sick (2021)
**Partnership**	
Number of partners	OECD (2018)
Types of partners (number)	OECD (2018)
Location of the partners	OECD (2018)
Prior collaborative experience	Guertler and Sick (2021)
Nature of partner involvement (identified previously by letters a through e)	OECD (2018)
**Process**	
Stage at which the collaboration begins	Cooper (1990; 2008)
Stages of partner involvement (all types)	Cooper (1990; 2008)
Number of stages of partner involvement	Cooper (1990; 2008)
**Results**	
Constraints	Guertler and Sick (2021)
Results	Guertler and Sick (2021)
Positive aspects of the collaboration	Guertler and Sick (2021)
Negative aspects of the collaboration	Guertler and Sick (2021)

SME, small- and medium-sized enterprise.

## Methodology

A multiple case study allows us to explore and learn from innovation collaboration in a real SME context (Yin, 2017). The innovation projects studied come from two different business contexts (pre-pandemic and early pandemic) and are representative of successful projects (new products developed and commercialized). Taking place over two time periods, the study also offers a longitudinal perspective of the phenomenon under study (Robson, 2011).

### Profile of SMEs and innovation projects studied

Based on a dozen SMEs, two Canadian manufacturing SMEs were selected for this longitudinal analysis. These two SMEs were able to carry out one product innovation project before the pandemic and another one at its beginning. These two SMEs were widely quoted in the media during the pandemic period. We had the opportunity to meet their managers to highlight the consequences of the crisis on their collaboration practices and their innovation processes, in a very particular period of their history.

In the four projects studied, new product ideas were developed through a process that included collaboration activities. The entire process could thus be documented. Table 2 presents some data on the SMEs and respondents in our study.

**Table 2.** SMEs’ and respondents’ data.

SME^2^	Number of employees	Sales (Canadian dollars $)	Industry	Position held by respondents	Education	Years of experience in innovation
Alpha	85	14 million	Food	CEO	MBA	28
				R&D manager	Engineering	15
Beta	265	40 million	Automotive (medical during the pandemic)	VP engineering	Plastics Technician	6
				Business development manager	Engineering Technician	2

SME, small- and medium-sized enterprise.

The innovation projects studied have different characteristics and are set in different contexts. At Alpha, the project studied before the pandemic concerns the development of refrigerated equipment for the food industry. At Beta, the project before the pandemic concerns the development of tooling for the automotive industry. In the context of the pandemic, the two projects put forward by Alpha and Beta concern the development of personal protective equipment intended to ensure the safety of thousands of employees in the food and medical sectors. Considered as working in non-essential sectors, according to the criteria established by the governments at the time, the two SMEs temporarily ceased their activities at the beginning of the pandemic. Their product innovation initiatives, however, allowed them to resume operations and re-employ most of their employees.

### Data collection and analysis

Data were collected through semi-structured interviews before the pandemic and at the beginning of the pandemic with two managers in each SME. Each interview lasted approximately 90 min and was conducted on the premises of the companies (in compliance with health regulations). At Alpha, interviews were conducted in December 2018 (pre-pandemic project) and July 2021 (project completed in March 2020). At Beta, interviews were conducted in March 2019 (pre-pandemic project) and September 2021 (project completed from March to June 2020).

With the respondents’ consent, the interviews were recorded and then transcribed. The interview patterns covered several topics: (1) description of the company and project, (2) innovation process, (3) organization, management, and structure of the innovation (resources, skills, collaboration). The data collection also included the consultation of nearly 100 pages of internal (e.g., innovation process map, tasks checklist, and specifications) and external (websites and press reviews) documents. Collecting data from a variety of sources and methods (interviews and secondary documents), known as triangulation, reduces the risk that a study's findings will reflect a single ‘viewpoint’ (Yin, 2017). This triangulation aims for a better understanding of the phenomena being studied and increases the fidelity of the data and conclusions (Fortin and Gagnon, 2016).

Analysis of the interview and document data followed the principles of thematic analysis (Paillé and Mucchielli, 2008): using NVivo software, the data were classified according to predefined themes based on the literature review and the categories and elements of comparison of the grid developed (cf. Table 1). These themes refer to the project (e.g., objectives and people involved), the innovation process (e.g., stages and activities), the partnership in innovation collaboration (e.g., types and nature) and the results (e.g., constraints and negative aspects). This analysis grid presented in Table 1 served as a framework for organizing and categorizing the empirical data collected. Data analysis enabled us to map innovation processes, for each of the periods studied, in each of the SMEs, and to compare the collaboration practices across the four projects studied. These maps were then transmitted to the respondents to ensure their validity, thus contributing to the richness of the data collected.

## Results

This section presents, first, the results obtained regarding the innovation processes of SMEs for the two periods studied. Second, the collaboration practices established by SMEs in the realization of their product innovation projects during these two periods are presented. Based on the analysis grid, we categorized the results following the categories and elements of comparison.

### Innovation process before and at the beginning of the pandemic

Table 3 provides the number of stages, activities, gates, and tasks identified in the innovation processes before and at the beginning of the pandemic. Differences are noted in the tasks before and at the beginning of the pandemic as well as similarities in the stages, activities, and gates of the SME processes. To understand these differences and similarities, we provide some explanations that highlight the specific context of the study (projects and SMEs) and the fact that the conclusions cannot be generalized to all SMEs and projects, which may not all share the same characteristics as those in our sample.

**Table 3.** Stages, activities, gates, and tasks in the SME innovation process.

SMEs	Stages (5)	Activities (13)	Gates (5)	Tasks
**Alpha (before)**	5	13	5 (#1 à #5)	46
**Alpha (beginning)**	3 (grouped)	5 (grouped)	3 (#1, #3, #5)	8
**Beta (before)**	5	13	5 (#1 à #5)	62
**Beta (beginning)**	3 (grouped)	5 (grouped)	3 (#1, #3, #5)	13

SME, small- and medium-sized enterprise.

Before the pandemic, the innovation processes of both SMEs followed a series of stages, activities, gates, and tasks that are similar to the original SGS model. The process distinctions between the two SMEs were more in the number of tasks, which were reduced at Alpha compared to Beta. These differences can be explained by the type of product developed, which at Beta required patents, unnecessary for Alpha. Alpha's closer proximity to its customers also resulted in a reduction in commercialization tasks. In comparison, Beta operates in a larger market requiring a commercialization plan, which increased the number of marketing tasks.

At the beginning of the pandemic, the innovation processes adopted by the two SMEs were characterized by their agility and speed. The stages and activities of the original SGS model are present, but some were grouped for speed. For example, some activities associated with the product development stage were performed simultaneously with those of the test and validation stage, which reduced their number. These groupings of stages and activities also reduced the number of gates from five to three and simple and few tasks were put forward in the process. In the context of the emergency associated with the pandemic, new products had to be developed and marketed quickly to protect the hard-hit food and medical employees. The higher number of tasks at Beta is explained by the fact that the SME had to adapt to a new sector of activity (medical), which led to the implementation of various tasks such as those associated with obtaining certifications from government medical authorities.

At Alpha, the innovation process adopted at the beginning of the pandemic took 5 days during which the new product (a protective gear made of plexiglas modules) was developed, tested and marketed at the local and provincial levels. Beta's innovation process took longer because of the approvals required before it could be marketed. It took 4 months (from March to June 2020) to develop the idea of a reusable face mask and market it locally and provincially.

This time frame is short compared to the typical time required to develop personal protective equipment for the medical sector in a non-pandemic context. (VP Engineering – Beta; *our translation*)

The changes made to the innovation process of the two SMEs at the beginning of the pandemic brought it closer to Stage-Gate Express while borrowing some of the principles of Agile Stage-Gate: iterations, feedback loops, and rapid exchanges between members of the extended team, including external collaborators. The collaboration partners also varied between the two periods studied. The adaptation to a new sector of activity (medical) for Beta also led to changes in partners.

### SMEs collaboration practices before and at the beginning of the pandemic

The collaboration practices before and at the beginning of the pandemic are depicted in Table 4. The comparison items are categorized as project, partnership, process, and results based on the grid developed from the literature.

**Table 4.** Collaboration in SMEs’ product innovation projects, before and at the beginning of the pandemic*.*

Categories and elements	Case Alpha–before	Case Alpha–beginning	Case Beta–before	Case Beta–beginning
**Project**				
**Project name^2^**	Food equipment	PPE – plexiglas modules	Automotive tooling	PPE – reusable masks
**Objectives^2^**	Better food preservation	Protection of individuals	Reduction of manufacturing time and number of parts	Protection of individuals
**Team involved (SME)^2^**	Multidisciplinary	Multidisciplinary	Multidisciplinary	Multidisciplinary
**Duration of the project^2^**	2 months	5 days	3 months	4 months
**Partnership**				
**Number of partners^3^**	4	6	4	7
Types of partners^2^ (number)*	1	2	1	3
**Location of the partners^3^**	Local/provincial	Local/provincial/national	Local/national/international	Local/provincial
Prior collaborative experience^2^*	Yes	Yes/no	Yes	Yes/no
**Nature of partner involvement^3^** **(a) through (e) previously identified)^3^**	(b) and (c) combined	(d)	(b) and (c) combined	(d)
**Process**				
**Stage at which the collaboration begins^4^**	Idea generation	At the beginning of the process	Idea generation	At the beginning of the process
Stages of partner involvement (all types)^4^*	Idea generation to commercialization	Idea generation to commercialization	Idea generation to commercialization	Idea generation to the last gate
Number of stages of partner involvement (all types)^4^*	5	3 (grouped)	5	3 (grouped)
**Results**				
**Constraints^3^**	Labour shortage	Labour shortage/business closures/supply difficulties	Labour shortage	Labour shortage/business closures/supply difficulties/regulations
**Results^3^**	Products commercialized	Products commercialized	Products commercialized	Products commercialized
**Positive aspects of the collaboration^3^**	Filling resources/project success	Filling resources/speed/project success	Filling resources/project success	Filling resources/speed/project success
**Negative aspects of the collaboration^3^**	Nothing reported	One partner removed from the project because it was not responsive enough	Nothing reported	Administrative burden of involvement with public sector partners

SME, small- and medium-sized enterprise.

Guertler and Sick (2021), ^3^OECD (2018), ^4^Cooper (1990, 2008).

**Figure 1.**
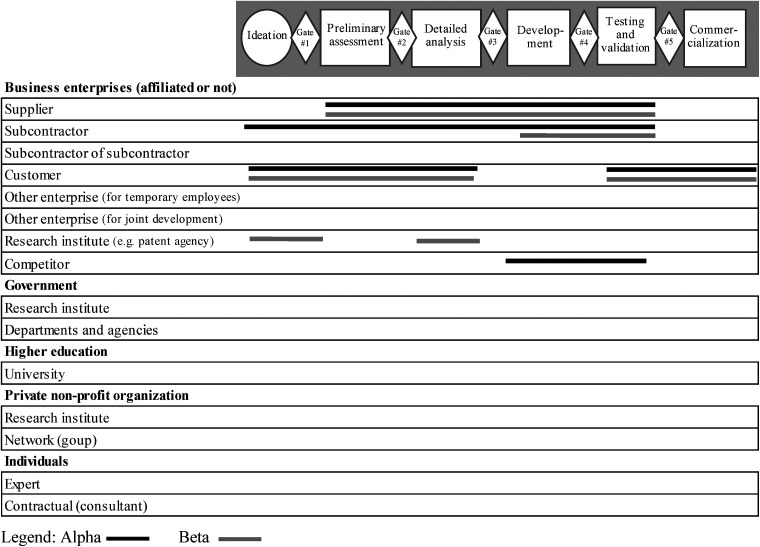
Collaboration partners in the SMEs innovation process, before the pandemic Legend: Alpha Beta.

**Figure 2.**
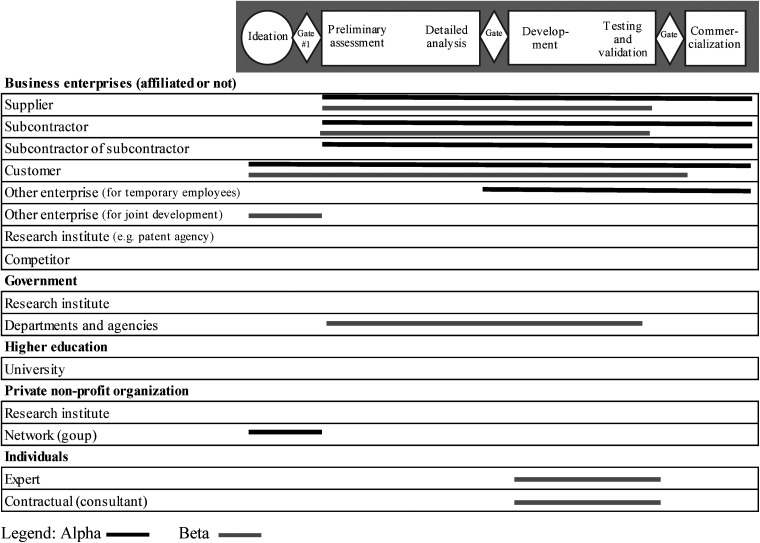
Collaboration partners in the SMEs innovation process at the beginning of the pandemic Legend: Alpha Beta.

* Figures 1 and 2 presented later in the document provide a detailed representation of the information.

Several points emerged from the results presented in Table 4. The diverse nature of the innovation projects undertaken by the two SMEs has resulted in the identification of both differences and similarities in their collaborative practices during the innovation process. We delve into these findings, providing explanations for the observed similarities and differences when comparing the projects conducted before and at the onset of the pandemic.

The location of the partners was mainly local at the beginning of the pandemic and expanded, as the borders between the provinces opened, to a more provincial and national market. The health crisis forced the closure of companies in many countries and limited the access of the SMEs to these external markets, whether for the supply or the sale of the new product developed. This became a constraint for the SMEs, which had to act mainly on the local market, more accessible. However, this proximity favoured a faster development of the new product, necessary to protect several thousand employees quickly.

As for the duration, it should be noted that the two projects carried out in both SMEs, at the beginning of the pandemic, were more radical than those carried out before the pandemic. In both cases, collaboration contributed to the success of the projects. The nature of the partners’ involvement was at level d where the new product is developed ‘as part of a collaborative agreement with other organizations with all parties contributing ideas and expertise’ (OECD, 2018: 135). This kind of collaboration made it possible to quickly fill in missing resources. The extended team involving partners was also formed earlier in the context of the pandemic because the participants had to quickly discuss the project, agree on its feasibility, and plan the actions to be taken next.

Several positive aspects emerged from the collaboration with partners in the development of new products while few negative aspects were identified for the two SMEs. The SMEs and their partners were invested in a ‘saviour’ mission that contributed to the fact that the extended teams involved wanted the new products to be developed, regardless of the effort required. There was also a need to diversify the enterprise's activities so that it could remain open in a situation that was very uncertain.

The foundation was to save Quebec (Province of Canada), to save people. We could not be open for our regular business. We could be open to develop protection strategies. (CEO – Alpha, *our translation*)

We were in a situation where at that time, the CERB (Canada Emergency Response Benefit^1^) did not exist. We did not know what was coming. Nobody knew what was going on. (CEO – Alpha, *our translation*)

In addition, engaging in product innovation allowed both SMEs to rehire their employees and hire new ones.

We were trying to generate as much value as possible to be able to call people back as quickly as possible to retain our workforce. Because it is pretty hard in our fields to get skilled labour. (VP Engineering – Beta, *our translation*)

Figures 1 and 2 provide a more detailed picture of the types of collaborative partners, their numbers, as well as the stages where collaboration occurred in the innovation process for both periods studied. For the sake of brevity, the innovation processes shown in these figures present only the stages and gates and do not detail the activities. Figure 1 shows that five stages and five gates were used by the SMEs before the pandemic. The three stages and three gates consolidated at the beginning of the pandemic are shown in Figure 2.

SME, small- and medium-sized enterprise.

SME, small- and medium-sized enterprise.

Figure 1 shows several similarities in terms of partners in the two SMEs, even though they operate in different industries and developed different new products. The SMEs collaborated with customers from the ideation stage to the detailed analysis stage. The goal was to solicit them several times during the process to understand their needs, evaluate the development of the idea with them and adjust quickly if needed. Customers were also involved in the testing and validation stage so that they could try out the new product as well as during the commercialization stage where their satisfaction was evaluated. Suppliers collaborated from the preliminary assessment stage through testing and validation. Managers wanted to plan the supply (quality, quantity and timing), which was complex because of the various possible variations of the product.

Certain distinctions can also be observed between the two cases studied. First, the subcontractors were more involved at Alpha. As early as the ideation stage, they were asked about the possibility of subcontracting certain components, which made it possible, from the first gate, to know whether or not it was possible to develop the idea. When their involvement was confirmed, they participated throughout the process until the testing and validation stage. At Beta, subcontractors were only involved in product development and testing because they only participated in certain basic components. Second, at Alpha, competitors had to collaborate in the purchase of specific components that the SME and subcontractors could not manufacture. This collaboration with competitors occurred during the product development and the testing and validation stages. Finally, Beta used a patent agent for the evaluation of the idea and the elaboration of solutions to be implemented from the ideation stage. Feedback was also given to the patent agent during the detailed analysis to confirm the technical specifications of the new product and the patent to be filed.

At the beginning of the pandemic, one of the main issues was supply difficulties. Most suppliers were closed due to government health restrictions. For both SMEs, this meant quickly contacting key suppliers and even finding new ones. International purchasing was also problematic. This supply problem and the urgency of developing the new product also left little room for in-house production. The two SMEs used their networks to find subcontractors. Alpha, among other things, contacted a group of business managers, right from the ideation phase, to target a local subcontractor who could respond quickly to its needs. Subcontractors were involved from the beginning of the process and some also collaborated with other subcontractors.

It became an extended team. We brought together local companies to develop the new product. Several companies (laboratories, manufacturers, specialists, designers) are involved in designing, developing and manufacturing the new product. (VP Engineering – Beta, *our translation*)

Customers were at the heart of new product development during the pandemic. In both SMEs, the problem was raised by them, and solutions had to be found quickly.

At the beginning of the process, everything was decided with the client. (R&D Manager – Alpha, *our translation*)

For Alpha, this collaboration continued through to commercialization. For Beta, the collaboration with the initial client ended before commercialization: this client was not involved in the commercialization of the new product to other target users.

Governmental authorities were also solicited by Beta to ensure that the product respected medical standards from the initial stages, right up to the tests performed on the products. This required the collaboration of an expert (researcher from an occupational health and safety research institute) and a consultant (quality specialist) in the product development and testing stages. Developing a new product in the context of a pandemic was:

A team effort with people from all over. A company could not have done this alone in such a short time frame. (VP Engineering – Beta, *our translation*)

It also helped make the innovation process more succinct, fast, and agile. Communication between the various partners was constant and necessary in solving problems, reducing technical risks, and confirming the continuity of the process.

The results, presented in Figure 1, also show that, before the pandemic, collaboration partners were limited to business enterprises (affiliated or not). During the health crisis, collaboration expanded to include other partners such as the government, non-profit organizations and individuals (Figure 2). Thus, in a crisis, there would be more variety in the collaborative partners to ensure success in product innovation projects.

## Discussion

In response to a crisis, some firms are likely to explore innovative solutions by seizing opportunities in new markets (Archibugi et al., 2013), which the two SMEs did. Despite operating in different industries and developing distinct new products, both similarities and differences appear in their innovation process and collaboration practices.

The product innovation processes studied indicate that both SMEs typically follow the stages and activities identified in the SGS theoretical model. These results are comparable to those obtained by Huang et al. (2002) with 276 Australian SMEs and by Leithold et al. (2015) with 49 German SMEs who showed that SMEs use many of the activities identified in the SGS. The availability of diverse technical resources and skills within both SMEs may explain the formal structure surrounding their innovation process. In times of crisis, when accelerated new product development is needed (Cooper, 2021), these resources and skills sometimes prove insufficient. Innovation collaboration can then be essential. This is consistent with the RBV theory that the choice of SMEs to engage in collaborative arrangements is partially based on access to complementary resources and skills (Arranz and Fdez. de Arroyabe, 2008).

Winch et al. (2021) find that changes to accepted project management practices result from extraordinary circumstances, such as the pandemic. Our results show that both SMEs developed product innovations with a higher degree of radicalness at the beginning of the pandemic with a shorter innovation process than before the pandemic. They both incorporate fewer tasks than in their initial innovation process and consolidate stages and activities for speed and agility. Edwards et al. (2020) note that a process that combines some of the stages/activities/tasks of SGS with agile approaches allows manufacturing SMEs to reduce product development times, get to market faster, and increase the success rate of their projects. Cooper (2021) advances that getting to market first (before competitors) can improve performance in market share, sales, and profit (Cooper, 2021), which we also noted among the SMEs in our study. Our results show that sales can be generated in SMEs by rapidly developing new products while firms that do not remain closed and inoperable.

The two cases analyzed also show that the nature of partner involvement, as proposed by OECD (2018) to evaluate the contributions of internal and external sources, evolves when time is short and the level of radicalness of the innovation increases. In regular times, SMEs combine sources (b and c), whereas collaboration becomes more prevalent at the beginning of the pandemic (d). Collaboration partners allow for more agility and speed in innovation processes since capabilities not available internally are obtained through external partners (El Hilali et al., 2020).

Comparing the collaboration practices in the innovation processes of the two SMEs, we notice distinctions between the two periods studied. Among other things, we note that suppliers and subcontractors are more involved in the pandemic context. When uncertainty is higher, their involvement can help SMEs confirm the feasibility of a project, reduce technical risks (Cooper, 2017) and access sufficient resources (Ledwith, 2000). This also helps to accelerate the manufacturing of components needed for new product development and improve the innovation process (Pierre and Fernandez, 2018), which helps to achieve success.

The involvement of customers is also crucial in both SMEs, where they are more present in the context of the pandemic. This proximity to customers assures SMEs that the products developed meet their needs, which has, as Morgan et al. (2019) indicate, a direct impact on the speed of product commercialization and the success of the projects. Thus, some partners, like subcontractors, are involved earlier or are involved longer, like customers, in the new product development project, depending on whether one is before or at the beginning of the pandemic.

Crises create an opportunity to innovate and collaboration plays an important role in accelerating the innovation process (Geurts et al., 2022). A rapid and effective response to crises is an important issue for SMEs due to their small size and limited resources (Eggers, 2020); therefore, collaboration is observed to act as a catalyst to achieve this. Collaboration partners were more numerous in the pandemic context in both SMEs and some of them had not collaborated before. This is consistent with the findings of Al-Omoush et al. (2022) that social capital, that is, the capabilities and resources obtained through external relationships rooted in the social networks of organizations to achieve shared goals, has a significant impact on innovation, collective intelligence, and the creation of sustainable competitive advantage in companies. The managers of the SMEs studied mentioned the importance of joint development in the success of their project. The need to collaborate with other enterprises of various kinds, research institutes and a grouping of managers, indicates that networks are more mobilized when a new product has to be developed and commercialized in a hurry. In that sense, Geurts et al. (2022) stipulate that crises generate the need to collaborate on innovation with multiple and diverse actors.

One might think that trust between partners emerges from mutual dependence, exacerbated by a sense of urgency generated by an unprecedented situation. SME managers can then become imbued with a rescue mission, beyond the SME's current activities (Geurts et al., 2022). Crises may foster tacit trust and mutual aid between partners who feel more in survival mode and invested in a mission; collaboration takes place on an informal rather than a formal basis.

## Conclusion, contributions, limitations, and further research

Based on multiple case studies of two manufacturing SMEs, our study shows that the health crisis changed product innovation processes and practices of innovation collaboration. The longitudinal approach of our study based on two periods shows how a crisis of the magnitude of COVID-19 influences the product innovation process and how collaboration with different, and sometimes new, partners favours its speed and success. A sense of urgency may prompt SMEs to quickly call on more collaborative partners and to involve them for longer in the process. With a simplified and accelerated innovation process, commercial success can still be observed in times of pandemic.

Regarding the theoretical contributions, this article combines two theoretical fields that tend to evolve in isolation: the innovation process (Cooper, 1990, 2008; Cooper and Sommer, 2016) and innovation collaboration (Guertler and Sick, 2021). Combining the product innovation process and the involvement of collaborative partners represents an innovative way of analyzing the changes induced by a crisis of innovation within SMEs. Also, empirical studies on innovation collaboration do not systematically mobilize theories to analyze the phenomenon (Cloutier and Amara, 2017; 2023), which our study addresses by mobilizing RBV theory.

In terms of managerial contributions, developing a new product in a hurry requires agility, related to the ability to adapt, which is important in the innovation process of SMEs. Collaboration in product innovation projects helps to bridge resources and skills and promotes the success of projects while contributing to the performance of SMEs. Innovation may allow SMEs to resume their activities when other businesses are closed, as is the case in the SMEs studied, by exploring the development of new products that differ from their usual ones. Collaborations with new partners, such as experts and government agencies, can be an essential element in the development of such new products. Our study highlights that the relational skills and networking of SME managers are important to identify partners and benefit from their contribution to product innovation projects, emphasizing the importance of social capital (Al-Omoush et al., 2022). Therefore, training to develop relational skills that promote collaboration is desirable. This study shows that public policymakers have a strong interest in promoting and encouraging collaboration, especially in crises, to foster innovation and continuity of SMEs.

As with any research, our study has limitations. First, our study focuses on changes in the practices of SMEs that successfully commercialized a new product at the beginning of the pandemic. It therefore does not enable us to identify the main obstacles faced by SMEs that did not attempt or succeed in developing new products during the pandemic. Also, the product innovation projects were not identical before and at the beginning of the pandemic in the two SMEs, which limits the extent of possible comparisons between the innovation collaboration process in normal times and in times of crisis, as well as the generalization of our conclusions to SMEs and projects that do not share the same characteristics. We can wonder whether the similarities and differences observed are due to other factors, namely the business model, the governance conditions, and the initial relational strategies. Questions also persist as to whether the observed changes in product innovation processes and collaboration practices are temporary or permanent.

These limitations may also form the basis of further research. It could, for example, be interesting to study whether the changes observed will persist when the context is equivalent to that which existed before the pandemic. Will innovation process and collaboration practices then be more in line with what they were before the crisis? Will SMEs have learned from the crisis and continue to collaborate in product innovation and accelerate their new product development projects by adopting a more streamlined process? A quantitative survey with a larger sample of SMEs to assess whether their innovation process and innovation collaboration practices have changed during the pandemic could also allow the characterization of profiles and the identification of the obstacles and levers to such innovation. Our results pave the way for further study on concepts such as organizational learning, organizational change, relational capital and crisis in product innovation by manufacturing SMEs.

In addition, this study focused on two manufacturing SMEs with a need to fill resources to innovate adequately. Future research could compare the collaboration profiles of SMEs with those of large firms to shed a different light on this phenomenon and gain a more complete picture. Also, comparing manufacturing firms with service firms would further explore the importance of context in collaboration practices. Finally, this empirical study is based on a sample of SMEs located in the province of Quebec, Canada. Both SMEs are therefore influenced by their context and researchers are encouraged to replicate this study in other regions and countries. Despite the limitations highlighted, this study contributes to the understanding of the adaptations made by SMEs to their innovation process and innovation collaboration in times of crisis.

This study attests to the high adaptability and resilience of the studied SMEs in the early stages of the pandemic, as they changed their processes and expanded their collaborative networks to rapidly commercialize new products. This indicates a potential for increasing the speed of innovation, which could ultimately shorten product lifetimes once again. This article hopes to join a multitude of other contributions to better understand how the pandemic has changed the innovation process and innovation collaboration.

## Notes

1. The Canada Emergency Response Benefit provided financial assistance to Canadian employees and self-employed individuals who were directly affected by COVID-19. Eligible individuals received a payment of $2,000 for a 4-week period (equivalent to $500 per week) between March 15 and September 26, 2020 (Government of Canada, 2022).

2. The names of the SMEs have been changed to ensure their anonymity.
